# The Onset of Diabetes During Transcranial Direct Current Stimulation Treatment of Anorexia Nervosa — A Case Report

**DOI:** 10.3389/fpsyt.2020.00040

**Published:** 2020-02-13

**Authors:** Tadeas Mares, Silvie Ceresnakova, Jakub Albrecht, Jozef Buday, Johana Klasova, Klara Horackova, Jiri Raboch, Hana Papezova, Martin Anders

**Affiliations:** ^1^ Department of Psychiatry, First Faculty of Medicine, Charles University and General University Hospital in Prague, Prague, Czechia; ^2^ First Faculty of Medicine, Charles University, Prague, Czechia; ^3^ National Institute of Mental Health, Klecany, Czechia; ^4^ Department of Internal Medicine of First Faculty of Medicine of Charles University and Military University Hospital, Prague, Czechia

**Keywords:** transcranial direct current stimulation, type 1 diabetes, anorexia nervosa, adverse effect, insulin resistance

## Abstract

The relationship between tDCS (transcranial direct current stimulation) and its influence on glycemia has been the aim of limited research efforts. Usually, the focus has been set on lowering the blood sugar level or interference with insulin resistance, but also the treatment of diabetic polyneuropathy and pain management. In this case report, we outline the development of hyperglycemia and the following onset of type I diabetes during a series of tDCS in a 24-year old Caucasian female patient treated with our research protocol (10 sessions; 2 mA; 30 min; the anode over F3; the cathode over Fp2) for anorexia nervosa.

## Background

Transcranial direct current stimulation (tDCS) is a type of neuromodulatory treatment which has recently received a considerable boom in popularity both in clinical and research applications. The method is economically reasonable, relatively safe and easy to use (1, 2).

A mild (usually 0,5–2 mA) electric current is utilized to primarily reach a subthreshold shift of resting membrane potentials towards hyperpolarization or depolarization in relation of current flow to axonal orientation (3, 4). This mechanism has been (depending on the current applied) shown to elicit excitatory or inhibitory response within affected brain areas (5, 6). Additionally, non-synaptic modulation contributes to the length of stimulation effect (7), while consecutive applications followed by a consolidation period is suggested to contribute to the accumulation of the effect and its expression (8). The effect of tDCS has been shown to encompass several different neurotransmitter systems — for instance dopaminergic (9, 10), seroroninergic (11), cholinergic (12), and glutamatergic (13, 14), thus affecting the functional connectivity, plasticity, and synchronization within several brain networks and topological organizations (15–17). Non-neuronal effect of tDCS on cells sensitive to electric fields has been proposed, but deeper understanding in this area is yet to be reached.

Adverse effects of tDCS are usually mild — redness and itching, mild fatigue or nausea being the most reported and mood changes and concentration difficulties much rarer (18).

Anorexia nervosa (AN) is a severe eating disorder characterized by food restriction, fear of weight gains, low weight and a strong desire to be thin. A distortion of self-perceived body image is present in many patients suffering from this condition and constitutes a serious health concern and a reason of frequent relapses (19).

The stimulation of dorsolateral prefrontal cortex (DLPFC) in patients suffering from AN has a few underlying theories. First, hyperactivity of right fronto-temporal area has been proven in this population by several imaging and EEG studies (20), hence the stimulation of L-DLPFC (or inhibition of R-DLPFC) is suggested to establish a balance between both DLPFCs. Other theories propose a positive effect of tDCS on maladaptive emotional regulation resulting in a decrease of food restriction or an effect on increased cognitive control (21, 22). One of the proposed mechanisms also includes a modulation of the mesolimbic pathway *via* the connection of DLPFC to striatum (23).

The studies focusing on tDCS in AN are limited and some of them are biased (24). Khedr et al. (25) suggested a potential effect of anodal tDCS on DLPFC in an open-label pilot study of 7 patients. Constanzo et al. (26) showed a significant increase of Body Mass Index (BMI) values in a group of patients suffering from AN compared to a group receiving family behavioral therapy. The improvement was noticeable at the end of the 6-week treatment and persisted even at a follow-up of 1 month (total sample size of 23 adolescents, anode over L-DLPFC, cathode over R-DLPFC). Similar results were reported recently by Strumila et al. in 2019 in 10 adult female patients (27).

In this article, we report a case of a female patient who developed type 1 diabetes (DM1) during research study of tDCS effect on self-perceived body-image in patients suffering from AN.

## Case Presentation

The patient, a 24-year-old, right-handed, Caucasian female, assistant nurse, with no previously diagnosed impairment of glucose metabolism was admitted to our Center for Eating Disorders due to her AN, which she suffered from 13 years of age. Current hospitalization was her 2nd stay in the department. She had been comorbidly diagnosed with posttraumatic stress disorder according to the ICD-10 (also valid under DSM-V). There was no history of type 1 diabetes in the family. Type 2 diabetes developed only in her grandmother (on mother's side) during the later stages of her life and was compensated on an insulin regimen. The patient was an everyday smoker of 2–3 cigarettes and an occasional drinker of 2 glasses of wine or 1 shot of hard liquor 2 times a week.

Upon admission, she was fully oriented in all spheres, with no signs of neurological deficit and unremarkable blood work (see **Table 1** for the initial lab values). She had a history of chronic bronchial asthma, gastritis and secondary amenorrhea for 6 months due to her eating disorder. Her medication consisted of 300 mg of trazodone (used irregularly due to her low compliance) and albuterol sulfate inhaler for her asthma. The initial BMI was 17.4 (h = 176 cm; w = 53.9 kg).

**Table 1:** Initial lab values of our patient. In some cases, SI unit conversion is shown.

Name	Value	Unit	SI conversion
Creatinin	68	µmol/l	
Urea	3.3	mmol/l	
Uric Acid	350	µmol/l	
Sodium (Na)	141	mmol/l	
Potassium (K)	hemolysis	mmol/l	
Chloride (Cl)	102	mmol/l	
Calcium (Ca)	2.47	mmol/l	
Glomerular Filtration Rate Lund-Malmo	1,375		
Estimated Glomerular Filtration CKD-EPI	1,802		
C-reactive protein (CRP)	<0,3	mg/l	(<2.86 nmol/L)
Bilirubin - direct	19	µmol/l	
Alanine Aminotransferase (ALT)	hemolysis		
Aspartate transaminase (AST)	hemolysis		
Alkaline phosphatase (ALP)	0.69	µkat/l	
Gamma-glutamyl transferase (GGT)	0.25	µkat/l	
Glucose	3.8	mmol/l	
Total Protein	77	g/l	
Albumin	50.8	g/l	(0.76 mmol/L)
White blood cells (WBC)	4.4	10^9^/L	
Reb blood cells (RBC)	5.11	10^12^/L	
Hemoglobin (HGB	154	g/l	
Hematocrit (HCT)	0.45	L/L	
Mean corpuscular volume (MCV)	88.1	fL	
Mean corpuscular hemoglobin (MCH)	1.8679	fmol	
Mean corpuscular hemoglobin concentration (MCHC)	212.2378	mmol/L	
Platelet count	168	10^9^/L	
Red cell distribution width (RDW)	12.6	%	
Platelet distribution width (PDW)	14.6	%	
Mean platelet volume (MPV)	11.6	fl	
Activated partial thromboplastin time (APTT)	27.6	s	
Prothrombin time (PT - INR)	1.13	INR	
Prothrombin time (PT - s)	13.2	s	

Her medication had been switched to 10 mg of escitalopram, 2.5 mg of olanzapine and her sleep had been augmented by 2 mg of bisulepin. She had been undergoing a specialized regime psychotherapeutic eating disorder programme with a positive weight gain of 1.8 kg in 2 weeks.

During the third week of hospitalization, she signed an informed consent and was enrolled in the randomized, double-blinded study focused on possible effects of tDCS on self-perceived body image. In the study we included patients between 18 and 65 years with a diagnosis of AN according to the ICD-10, we excluded patients with a history of brain injury, epileptic paroxysm, migraines or with metallic object within the neurocranium. We used Anamorphic Micro computer application to objectify the patient's perceived body image distortion. The patient initially overestimated her bodily size by 122% and desired a size of 101% compared to her objective proportions (objective dissatisfaction), which constituted 83% of her perceived size (subjective dissatisfaction) suggesting a gross distortion of body image congruent with the diagnosis and possibly leading to her rehospitalization.

**Figure 1:**
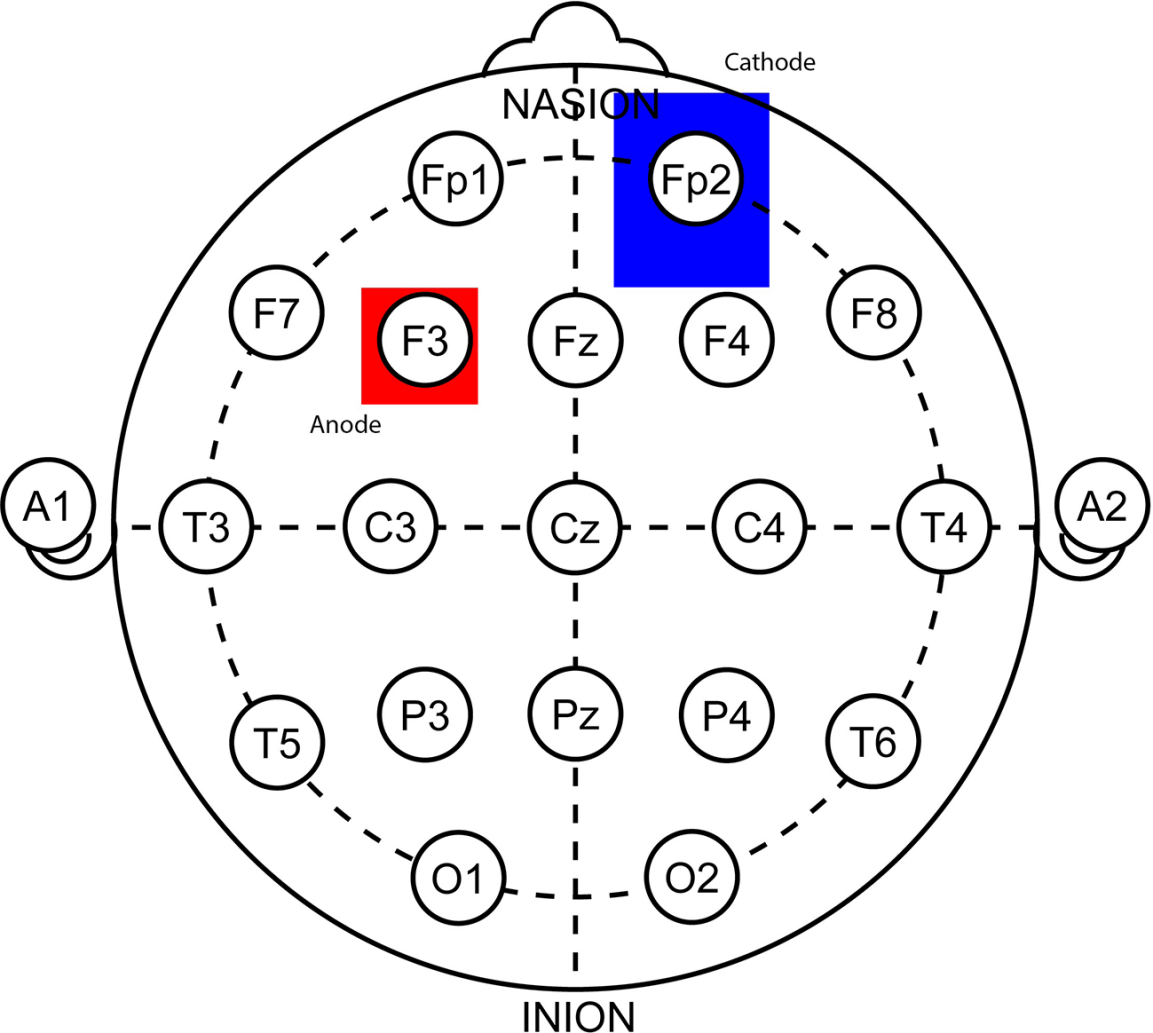
A depiction of electrode positioning in a 10–20 EEG system. The anode (red, 5x5 cm) over the left dorsolateral prefrontal cortex (F3) and the cathode (blue, 6x8,5 cm) over the right orbitofrontal region (Fp2). It was ensured there is at least 8 cm between these electrodes in order to prevent shunting.

She had been scheduled to undergo 10 sessions of 30min, 2mA anodal stimulation of the left DLPFC (F3, Current Density of 0.571 A/m^2^) with cathode over the right orbitofrontal region (Fp2) (**Figure 1**). We used the HDCstim portable programmable direct current stimulator (manufactured by Newronika s.r.l.) with conductive silicone electrodes (anode 5x5 cm, cathode 6x8,5 cm) covered by hydratable holding bags in plant cellulose soaked in saline (0.9%).

On the second day of stimulation, the patient started referring to blurred vision, but she attributed it to her fatigue. The physical and neurological exam was unremarkable, and the patient did not wish to pursue any further examination.

On the 6th day of stimulation, she complained of recurring problems with eye accommodation and increasing fatigue, an onset of polydipsia resulting in polyuria was noticed. These symptoms were considered a part of her eating disorder. Nevertheless, an ophthalmological appointment was set up. At the beginning of the 7th day of stimulation, the research team was fully informed about the extent of the aforementioned symptoms and the principal investigator decided to stop any further application until the patient ´s state gets thoroughly examined and resolved, concurrently it was decided to unblind the study. We found out, that a real (non-sham) stimulation was used in the patient.

On the 8th day the symptoms of polyuria, polydipsia, and blurred vision had worsened, the ophthalmologist prescribed corrective lenses and suspected the unusual findings to be of psychogenic etiology.

Extended blood work and an internal exam were ordered showing a fasting glucose level of 39 mmol/l (702 mg/dl). The patient became disoriented, confused and subdelirious, nauseous and referring to abdominal pain. She was immediately rushed to the acute internal ward to properly manage the hyperglycemic crisis.

Further diagnostic tests showed markedly decreased C-peptide levels and negative DMAP (Diabetes Mellitus Autoantibody Panel), which met the ICD-10 criteria for a diagnosis of DM1. Her olanzapine medication had been immediately discontinued and switched to quetiapine. The ultrasound exam ruled out any gross pancreatic pathology and in the next week the blood glucose level normalized on an intensified insulin regimen of 3 units of aspart 3 times a day and 2–4 units of glargine overnight.

During our follow-up the perceived size constituted 123.5% compared to reality, the objective dissatisfaction reached 116% and the subjective dissatisfaction was 94%.

## Discussion

All criteria for type 1 diabetes were met, however, one excluding criterion might have been neglected. There might have been an underlying condition in the form of tDCS. In the current literature, there are a few studies and case reports indicating a possible effect of tDCS on the modulation of the blood sugar levels.

Wardzinski et al. (28) successfully increased the systemic glucose tolerance and cerebral high-energy phosphate content by utilizing the anodal stimulation of the right motor area (C4) with the cathode over Fp1. A protocol of 2 sessions of 1 mA tDCS lasting 20 min was used. The sessions were divided by roughly 115 min. The study was done on 15 healthy, adult male volunteers in a cross-over, single blinded design with a pause of at least 2 weeks between the sham and the real tDCS.

Kistenmacher et al. (29) was able to decrease the blood sugar levels in healthy individuals by insulin-independent mechanism even 8 days after the stimulation. A protocol of single tDCS (1 mA, 20 min, anode over C4, cathode over Fp1) was used in 14 healthy, adult males in a single-blinded cross-over design with the real procedure separated by at least 2 weeks from the sham.

We acknowledge the proven paradigm of tDCS, that every montage is unique, inducing a different set of effects. Even though we utilized an anodal stimulation of the left hemisphere with cathode over the right (an opposite approach of the aforementioned studies), our electrode placement was different and thus we cannot make any claims regarding the possible opposite effect of our protocol.

There is a large number of studies utilizing a montage similar to ours. To our knowledge, there has not been any described case of insulin/blood sugar related adverse effects. Currently, there is no evidence suggesting that a tDCS protocol could increase the levels. On the contrary, studies exploring the blood sugar levels in this montage have never been done.

There may have been other contributing factors. Primarily we considered a pre-existing impairment of glucose metabolism. On the contrary, the patient was undergoing a regular blood sugar checkup at her GP and her initial bloodwork did not suggest any irregularities in glycemia. We have to admit we did not perform nor did we require the diabetes test panel initially, since the patient had never manifested any symptoms and there was no suspicion of the possible development of diabetes before.

There is also the question of the diabetogenic adverse effects of olanzapine, which has already been established as a strong prodiabetogenic agent. The correlation between the dosage and relative risk for the development of diabetes has not been proven. The actual dosage of 2.5 mg is widely considered safe (30). Olanzapine-induced diabetes usually improves with the discontinuation, but the condition persists and the patient remains on a diabetic diet with a stable insulin regimen. Moreover, according to the diabetologic assessment, the patient did not suffer from increased insulin resistance for 3 months after the episode, which would also be a point against this hypothesis.

We also considered a possible increase in the saccharide intake. The patient consumed irregularly a maximum of 4,000–5,000 kilojoules (956–1195 calories) a day (with a prevalence of sugars) with certain days of only 2 instant soups a day. This regimen lasted for at least a month prior to the hospitalization. The food intake was increased after the admission and reached 10,000 kilojoules (2,390 calories) a day consisting of roughly 60% sugars, 17% proteins, and 23% fats, which is a standard diet in our eating disorder programme (see **Table 2** for a more comprehensive summary of food intake and other lifestyle factors). The change in the diet contributed to a positive weight gain and could have certainly contributed to the increased blood sugar levels, but we have to note that there were no symptoms of hyperglycemia prior to the stimulation even though the patient ´s food intake was increased for two weeks by that time.

**Table 2:** A summary of food intake, smoking habits, alcohol consumption, and sleep in the patient.

Time period	Food Intake	Smoking	Alcohol consumption	Sleep
Prior to the admission (3 months)	a maximum of 4,000 - 5,000 kJ (956–1,195 calories) a day, with intermitent episodes of food restriction (2 instant soups a day)	2–3 cigarettes a day	2 glasses of wine/1 shot of hard liquor 2 times a week	not content with her sleep, frequent nightmares, dyssomnia
During the hospitalization (1 month)	cca 10,000 kJ (2,390 calories) of high-energy hospital diet consisting of approximately 60% sugars, 17% proteins, and 23% fats	2–3 cigarettes a day	0	content with her sleep, dyssomnia compensated
After the diagnosis of DM1	6,000–7,000 kJ (1,434–1,673 calories) of diabetic diet with a limitation of 200 mg sugars a day	2–3 cigarettes a day	0	no complaints with regards to her sleep patterns

We feel the case uncovered certain inadequacies in the internal communication within our research team, which has been worked on and improved substantially since then. It would have certainly been advisable (in accordance with Good Practices in Research) to stop the stimulation upon the occurrence of the first adverse effects and to wait upon their resolution. We kindly ask the reader to pay attention to these feedback mechanisms in their own research.

As for the changes in the Anamorphic Micro programme (31), the patient's self-perceived body image remained without any significant changes. On the contrary, we measured a noticeable improvement in her objective and subjective dissatisfaction, which led to an increased tendency to build up weight. The results could be interpreted as an improvement of emotional processing of her body-image.

Interestingly, the patient started menstruating again after several tDCS applications, but we cannot be certain (as with the changes in the Anamorphic measurements) whether it was caused by the slight weight gain, the metabolic changes caused by the DM1 or the stimulation itself.

## Conclusion

The extent of tDCS effect on glucose metabolism is vastly unexplored. To our knowledge, there has never been a described case of hyperglycemia and the onset of DM1 coinciding with the application of tDCS. Direct current stimulation was suggested by some authors to decrease the blood sugar levels, but studies focusing on the opposite have understandably never been done. Patients suffering from AN are a specific population and caution may be worthwhile when using tDCS. We also showed an improvement of our patient's self-perceived body dissatisfaction during tDCS resulting in a positive weight gain, but further research efforts are required to properly evaluate the role of the stimulation itself as well as to investigate the possible influence of tDCS on the glucose/insulin metabolism.

## Data Availability Statement

All our data regarding the case report are included in the main body of text. The datasets generated for the whole research study will be published and made publicly available in the future upon their completion.

## Ethics Statement

The studies involving human participants were reviewed and approved by ETHICS COMMITTEE of the General University Hospital, Prague. The patients/participants provided their written informed consent to participate in this study. Written informed consent was obtained from the individual(s) for the publication of any potentially identifiable images or data included in this article.

## Author Contributions

TM and SC performed the stimulation as part of their research and wrote the case report. JA provided senior expertise in stimulation techniques. JB contributed with literature review for the project. JK provided a diabetologic expertise in the project. KH provided an expertise on eating disorders. JR provided expert psychiatric supervision. HP is the head of the research and provided expert supervision. MA organized the stimulational assets used in the project and ensured their expert supervision.

## Funding

The study was supported by the Charles University Project GA UK No.104119; MH CZ – DRO VFN64165; Q27/LF1; NPU I (LO1611) and AZV 15-31538A.

## Conflict of Interest

The authors declare that the research was conducted in the absence of any commercial or financial relationships that could be construed as a potential conflict of interest.
